# ILCOR’s revised Covid-19 defibrillation recommendation requires a new approach to training

**DOI:** 10.1186/s13049-020-00804-y

**Published:** 2020-11-07

**Authors:** John A. Stewart

**Affiliations:** 9407 Linden Ave. N, Seattle, WA 98103 USA

**Keywords:** In-hospital cardiac arrest, In-hospital resuscitation, Covid-19, Defibrillation, Training

## Abstract

In-hospital resuscitation practices have changed by necessity in the Covid-19 era, principally due to precautions intended to protect caregivers from infection. This has resulted in serious delays in resuscitation response.

ILCOR has recently modified its guidelines to separate defibrillation from other interventions, recognizing that shock success is extremely time-dependent and that defibrillation poses relatively little risk of Covid-19 transmission. The new recommendation calls for sending one caregiver into the isolation room in order to initiate bedside monitoring and defibrillate if indicated, while the code team is donning their personal protective equipment. Implementing this change requires focused training in that specific role. This can be accomplished by intensively training a subset of clinical staff to assume the responsibility and act without hesitation when a code occurs.

Focused defibrillation training promises to avoid compromising the care of patients experiencing tachyarrhythmic arrests in the setting of Covid-19. Such a training program might even result in better survival than before the pandemic for this subset of patients.

## Background

The Covid-19 pandemic has stimulated changes to many aspects of healthcare, including the response to cardiac arrests both outside of and within hospitals. Potential transmission of Covid-19 to caregivers is a central concern, and personal protective equipment (PPE) is used to protect caregivers from infection. In hospitals, the basic protections are droplet and contact isolation of suspected Covid-19 patients, with airborne precautions recommended for aerosol-generating procedures (AGPs) [1]. These precautions necessarily cause treatment delays.

With their recent revision, the International Liaison Committee on Cardiopulmonary Resuscitation’s (ILCOR’s) guidelines reflect these PPE guidelines but also specifically identify defibrillation as unlikely to generate aerosols, consequently recommending that hospitals “consider attempting defibrillation [for patients with tachyarrhythmic arrests] before donning personal protective equipment (PPE) for aerosol generating procedures” and before starting chest compressions [2] —echoing an approach developed in 2003 in response to the severe acute respiratory syndrome coronavirus (SARS-CoV) crisis [3]. This is a change from the former goal (rarely achieved in practice) of starting chest compressions and monitoring/defibrillation simultaneously.

Even before the Covid-19 pandemic, there were good reasons to focus attention and resources on rapid defibrillation. Defibrillation is often thought of as just one of many elements of emergency cardiac care, but it is in fact the only definitive treatment for any type of cardiac arrest--and success decreases very rapidly with time. Some 20 years ago, American Heart Association and ILCOR resuscitation leaders acknowledged the problem of delayed defibrillation and began promoting automated external defibrillation in hospitals [4], resulting in the widespread adoption of dual-mode (automated and manual) defibrillators by hospitals. That approach failed to improve survival (no change for tachyarrhythmic arrests, lower survival for other arrhythmias) [5], and interest in the problem waned afterward.

## Main text

The rationale for ILCOR’s revised defibrillation recommendation is simply that defibrillation success is clearly very time-dependent [6] and donning PPE necessarily causes delays in treatment. Since defibrillation is not thought to produce significant aerosols (i.e., is not an AGP), it should not be delayed to put on full PPE. Instead, one person should immediately take the monitor/defibrillator into the room, initiate bedside cardiac monitoring, and defibrillate if indicated, while others are donning their PPE. An additional consideration is that defibrillation can be done from more than 2 m distant from the patient, further decreasing transmission risk [7].

This approach can complicate resuscitation efforts. If several caregivers are immediately at the scene, as is commonly the case, the caregiver designated to go first into the isolation room should be both willing to perform the role and proficient in defibrillation. Assigning that role on the spot will almost certainly involve a significant delay. One alternative would be to assign resuscitation team roles in advance, but there would be no way to ensure that the person designated to defibrillate could be on the scene quickly. In addition, the act of defibrillation can be intimidating for a number of reasons (the sudden call to perform in a life-or-death situation; fear--albeit largely groundless--of injury to oneself, the patient, or others), and concerning delays have been documented repeatedly in clinical simulations [8, 9]. Add to these factors the challenge of entering the room and being expected to perform alone, without full PPE, and the task can appear quite daunting.

Exploration of another approach is warranted—especially now, with the current Covid-19 pandemic. A cadre of nurses or other caregivers (perhaps one in four) could be trained intensively for one specific role in every code: to provide initial monitoring and defibrillation as quickly as possible [10]. For Covid-19 arrests, they would automatically be designated as the first to enter the room, perhaps identified by distinctive ID badges, while other caregivers donned their PPE. They could defibrillate if indicated—saving precious minutes—and withdraw as others arrived, whether to don their own PPE and rejoin the team or to resume other duties. If defibrillation was successful, the code would likely be shortened, thus decreasing the risk of Covid-19 transmission to caregivers while also giving the patient a decent chance to survive.

Most resuscitation interventions assume a supine patient, but Covid-19 patients are frequently positioned prone. In the event of a code they must be turned over for most emergency interventions, requiring multiple staff and additional delay [11]. However, defibrillation can easily be done before this major effort, with electrode pads applied in bi-axillary or postero-lateral (left mid-axillary line and right scapula) position [12].

Focused defibrillation training promises to decrease confusion and delay in Covid-19 resuscitation efforts. It may also help with some of the hard decisions caregivers face about limiting or withholding resuscitation efforts [13]. Defibrillating first could provide a reasonable endpoint for efforts in some cases, given that survival from other presenting arrhythmias is very low [14].

## Conclusion

Resuscitation guidelines are by necessity changing in the setting of Covid-19, but delivery of defibrillation need not be compromised and might even be improved by focused defibrillation training. Caregivers would not be exposed to significant additional risk, and patients with tachyarrhythmic arrests would be given their first and best chance of survival.

## Data Availability

Not applicable.

## Abbreviations

PPE: Personal protective equipment
AGP: Aerosol generating procedure
ILCOR: International liaison committee on cardiopulmonary resuscitation
